# Advanced Monte Carlo for Acquisition Sampling in Bayesian Optimization

**DOI:** 10.3390/e27010058

**Published:** 2025-01-10

**Authors:** Javier Garcia-Barcos, Ruben Martinez-Cantin

**Affiliations:** Instituto Universitario de Investigacion en Ingenieria de Aragon (I3A), Universidad de Zaragoza, 50018 Zaragoza, Spain; jgbarcos@unizar.es

**Keywords:** Bayesian optimization, Gaussian process, MCMC

## Abstract

Optimizing complex systems usually involves costly and time-consuming experiments, where selecting the experiments to perform is fundamental. Bayesian optimization (BO) has proved to be a suitable optimization method in these situations thanks to its sample efficiency and principled way of learning from previous data, but it typically requires that experiments are sequentially performed. Fully distributed BO addresses the need for efficient parallel and asynchronous active search, especially where traditional centralized BO faces limitations concerning privacy in federated learning and resource utilization in high-performance computing settings. Boltzmann sampling is an embarrassingly parallel method that enables fully distributed BO using Monte Carlo sampling. However, it also requires sampling from a continuous acquisition function, which can be challenging even for advanced Monte Carlo methods due to its highly multimodal nature, constrained search space, and possibly numerically unstable values. We introduce a simplified version of Boltzmann sampling, and we analyze multiple Markov chain Monte Carlo (MCMC) methods with a numerically improved log EI implementation for acquisition sampling. Our experiments suggest that by introducing gradient information during MCMC sampling, methods such as the MALA or CyclicalSGLD improve acquisition sampling efficiency. Interestingly, a mixture of proposals for the Metropolis–Hastings approach proves to be effective despite its simplicity.

## 1. Introduction

Solving engineering and scientific problems often requires the use of complex computer models, setting up expensive prototypes, resource-intensive experimentation, or tuning the best parameters of a deep learning model. Identifying the best configuration for these processes requires evaluating various options with a trial-and-error approach. Given the high cost and time associated with these tasks, it is essential to achieve high sample efficiency. Although traditional optimization methods and evolutionary algorithms can be effective in finding the optimal configuration, they may not always be as sample-efficient as certain expensive contexts require. Carefully selecting which experiments to conduct is crucial. Bayesian optimization (BO) [1,2,3] is a method that helps streamline the process of selecting the most promising experiments and identifying optimal solutions as efficiently as possible.

BO builds a probabilistic surrogate model that approximates the unknown target function from observations. Then, an acquisition function based on the surrogate model is used to find which point should be observed next, balancing exploration and exploitation to achieve sample efficiency. This process is intrinsically sequential, and the standard BO formulation is limited to single experiment evaluations.

**Figure 1:**
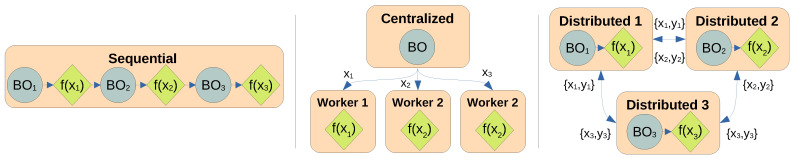
Types of parallelization schemes in Bayesian optimization (BO). The left side shows the sequential node, which is the standard BO approach, where BO computations and sample evaluation are strictly sequential and usually carried on a single node. The middle shows a diagram of parallel BO, with one node carrying the BO computations and multiple workers evaluating samples in parallel, including asynchronous evaluations. The right shows the fully distributed approach, where each node shares data asynchronously with other nodes while being capable of operating independently of each other. The lack of centralization and synchronization ensures the highest resource utilization possible.

Alternatively, many costly problems can be performed cheaper and more efficiently using a parallel BO strategy that enables parallel experimentation, such as chemical experiments in a wet lab for drug discovery [4], multi-agent experiments [5], and autoML in a cluster [6]. Parallel BO can be achieved with a batch setting [7,8], where multiple experiments can be proposed to be carried out in parallel, including asynchronous execution [9]. These BO parallel approaches are centralized, with a single central node typically carrying out the bulk of the BO computation of updating the surrogate model and proposing the batch of experiments, which will be evaluated by many worker nodes. This hinders maintaining privacy in federated learning [10] and resource utilization when experiments are carried out with high-performance computing [11]. To solve this, BO can be adapted to work in an asynchronous decentralized fashion [12,13]. This fully distributed workflow enables the experiment to be carried out even if there is a partial failure in the distributed network since data is shared asynchronously and new experiments can be proposed independently. Figure 1 compares them.

A simple approach to achieving a fully distributed BO is replicating the BO algorithm across multiple nodes, sharing data asynchronously and, to prevent different nodes from suggesting the same exact experiments, relying on sampling approaches such as Thompson sampling [4] and Boltzmann sampling [13] with different random seeds. In particular, Boltzmann sampling has some interesting properties, such as being able to operate with existing well-understood and tested acquisition functions and offering better robustness to modeling inaccuracies [14]. Boltzmann sampling also requires setting a temperature parameter that depends on the acquisition function values. However, there is high variability in the acquisition values, since they can change considerably as the optimization progresses. This means that, in some cases, it is not feasible to select a temperature parameter that performs well throughout all the optimization steps. Since direct sampling is not possible due to not having access to the explicit form of the quantity we are sampling from, Boltzmann sampling relies on using Markov chain Monte Carlo (MCMC) methods [15], which enables efficient sampling in higher dimensions of unnormalized probabilities.

In this paper, we propose an alternative formulation of Boltzmann sampling by instead proposing to sample directly from the acquisition function, which avoids having to set a temperature parameter and has better performance across benchmarks. We analyze different MCMC methods found across the literature for performing acquisition sampling and evaluating the resulting Bayesian optimization performance. In particular, we study the main challenges of applying MCMC to acquisition sampling: (1) we deal with a constrained domain defined by the search space of BO, (2) numerical instabilities of the acquisition function prevent proper, accurate representation of the target density, and (3) acquisition functions are highly multimodal, and sampling them is challenging for MCMC methods.

### 1.1. Bayesian Optimization

Bayesian optimization [16] is a sequential optimization method that aims to optimize expensive functions in a sample-efficient manner. It employs a *probabilistic surrogate model* p(f) that learns the characteristics of the function that we aim to optimize, capturing the uncertainty and providing predictive distributions, which is then used by an *acquisition function* α(x,p(f)) that rates the next queries. Formally, it aims to optimize an unknown objective function f:X→R over a domain $X\subset R^{d}$, by carefully selecting the queries of the function to efficiently identify the optima $x^{*}$. At iteration *t*, for readability, all previously observed values as $y_{1:t}$ at queried points $x_{1:t}$ are used to construct a probabilistic surrogate model $M_{t}=p\left(f|y_{1:t},x_{1:t}\right)$. For readability, our notation will omit the subscript for observed data, defining $X=x_{1:t}$ and $y=y_{1:t}$, with a set of points and values defined as $D_{1:t}=\left\{x_{1:t},y_{1:t}\right\}$.

This probabilistic surrogate model frequently takes the form of a Gaussian process (GP). It is a distribution over functions with a continuous domain, which is defined by GP(μ(·),k(·,·)), where μ:X→R is the mean function and k:X×X→R is a covariance function or kernel that has kernel hyperparameters θ. For the experiments, we assume μ(·)=0, and the kernel *k* is the Matérn kernel 5/2, defined as

(1) $$k_{\mathrm{Matérn}}\left(x,x^{\prime }\right)=\sigma_{f}\left(1+\frac{\sqrt{5}\left|x-x^{\prime }\right|}{\ell }+\frac{5{\left(x-x^{\prime }\right)}^{2}}{3\ell^{2}}\right)\exp\left(-\frac{\sqrt{5}\left|x-x^{\prime }\right|}{\ell }\right)$$

with parameter *ℓ* as the lengthscale and the scaler parameter $\sigma_{f}$ as the amplitude parameter. These are the hyperparameters of the GP that we will need to adjust $\theta =\{\ell ,\sigma_{f}\}$. Note that all methods in this paper are independent of mean and kernel choice.

Having decided both the mean μ(·) and kernel k(·,·) functions, we can now use the GP posterior model to make predictions at query points $x_{q}$, which are normally distributed $y_{q}∼N\left(\mu \left(x_{q}\right),\sigma^{2}\left(x_{q}\right)\right)$. This predictive distribution can be computed in closed form:

(2) $$\begin{array}{cc} \mu (x_{q}) & =\mu \left(x_{q}\right)+k{\left(x_{q}\right)}^{T}K^{-1}y \end{array}$$

(3) $$\begin{array}{cc} \sigma^{2}\left(x_{q}\right) & =k\left(x_{q},x_{q}\right)-k{\left(x_{q},x\right)}^{T}K^{-1}k\left(x_{q},x\right) \end{array}$$

where the covariance matrix of the observed data points is K=k(X,X) for noiseless observations, or, when modeling the noise in the observations y=f(x)+ϵ, the kernel becomes $K=k\left(X,X\right)+\sigma_{n}^{2}I$, with $\sigma_{n}^{2}$ being the Gaussian likelihood noise variance.

With the GP as our model M, we can now define the overall algorithm for Bayesian optimization, as shown in Algorithm 1. First, it requires some initial data. Then, it involves sequentially updating the model M with the current observations, choosing a query with a selection strategy and performing the observation of said query. This is repeated until the budget *T* is exhausted, in which it returns the best-observed value $\left\{x^{*},y^{*}\right\}$. This is a valid recipe for sequential and batched parallel selection strategies, including asynchronous evaluations [9], which can be done by fantasizing about possible values of pending observations.
**Algorithm 1** Bayesian Optimization**Require:** Budget *T*  1: $D_{1:p}$ ← Query *p* initial points based on LHS  2: **for**
t=p…T
**do**  3:    $M_{t}$ ← Update surrogate model with $D_{1:t}$  4:    $x_{q}$ ← Selection strategy using $M_{t}$▹ See Section 2  5:    $D_{1:t+1}$ ← $D_{1:t}\cup \left\{x_{q},f\left(x_{q}\right)+\epsilon \right\}$▹ Observe query $x_{q}$  6: **end for**  7: return $\leftarrow \left\{x^{*},y^{*}\right\}=D_{i}\;s.t.\;i=\arg\max_{i}y_{i}$

Updating the model involves estimating the Gaussian process hyperparameters θ, typically by using the log marginal likelihood. The marginal likelihood or evidence p(y|X,θ) is the probability of the observed data given the input locations X and the hyperparameters θ. Log marginal likelihood is expressed as

(4) $$\log p\left(y|X,\theta \right)=-\underset{\mathrm{Data}\;\mathrm{fit}}{\underset{︸}{\frac{1}{2}{\left(y-\mu (X)\right)}^{T}K^{-1}\left(y-\mu (X)\right)}}-\underset{\begin{array}{c} \mathrm{Complexity}\\ \mathrm{penalty} \end{array}}{\underset{︸}{\frac{1}{2}\log\left|K\right|}}-\underset{\mathrm{Normalization}}{\underset{︸}{\frac{n}{2}\log2\pi }}$$

with the data term assessing how the model explains the observed data; the complexity penalty term discourages complexity that might overfit the data and normalization constant to produce a valid probability distribution. We will employ an *empirical Bayes* approach, where the hyperparameters are set to the most likely value, that is the maximization of the log marginal likelihood. This optimization can be done with numerical methods such as gradient descent or gradient-free methods such as L-BFGS.

Bayesian optimization requires some initial data $D_{1:p}$ so that we can start making predictions and select the next queries. Therefore, the optimization is initialized with *p* evaluations from a low discrepancy sequence, such as a Latin Hypercube sampling or a Sobol sequence.

### 1.2. Distributed BO

Most parallel versions of Bayesian optimization use a form of batch BO, where in each step, a set of query points are generated following a specific acquisition method that guarantees diversity and avoids repetitions on the query set [7,9,17,18]. As shown in Figure 1, these methods rely on a central node that has access to all the information and guarantees the diversity of query points. Furthermore, batch BO requires that queries are generated synchronously, which might be a problem in applications where different experiments might vary in time [9]. Instead, here we propose a fully distributed BO method as shown in Figure 1, which does not have any centralized node; all computations can be performed independently at each node within the distributed system, where diversity is built by construction. An advantage of this structure is that queries can be generated asynchronously as soon as new resources are available to perform experiments.

The BO algorithm from Algorithm 1 can be modified to a fully distributed system, as shown in Algorithm 2, where it shows the operations of a single node of the distributed system. Compared to the sequential BO algorithm, the only changes required are that the evaluation of initialization data is distributed across nodes, that nodes broadcast and receive their latest observations, and, most importantly, that the selection strategy is compatible with being fully distributed, that is, it ensures that the nodes are capable of observing different queries even if they have exactly the same data $D_{1:t}$ and model M, such as in Thompson sampling [4,12] or Boltzmann sampling [13,14].
**Algorithm 2** Distributed Bayesian Optimization Node**Require:** Budget *T*  1: $D_{1:p/N}$ ← Query p/N initial points based on LHS  2: Broadcast ← $D_{1:p/N}$▹ To all other available nodes  3: **for**
t=p…T
**do**  4:    $D_{1:t+1}$ ← $D_{1:t}\cup \left\{x_{\mathrm{other}},y_{\mathrm{other}}\right\}$▹ Collect other node data if available  5:    $M_{t}$ ← Update surrogate model with $D_{1:t}$  6:    $x_{q}$ ← Selection strategy using $M_{t}$  7:    $D_{1:t+1}$ ← $D_{1:t}\cup \left\{x_{q},f\left(x_{q}\right)+\epsilon \right\}$  8:    Broadcast ← $\left\{x_{q},f\left(x_{q}\right)+\epsilon \right\}$▹ To all other available nodes  9: **end for**10: Sync data with other nodes▹ So that all nodes return the same optimum11: return $\leftarrow \left\{x^{*},y^{*}\right\}=D_{i}\;s.t.\;i=\arg\max_{i}y_{i}$

Figure 1 shows how the nodes only need to broadcast their queries and observed values $\left\{x_{i},y_{i}\right\}$ to have access to all the information available at each moment, which requires minimal communication bandwidth. However, each node can perform optimization independently. Therefore, communication can be asynchronous and resilient to network failures, as the order of queries and observations does not affect the process, and nodes can keep working when isolated from the rest of the network if needed.

## 2. Selection Strategies

In this section, we present several acquisition function methods to generate new queries. Traditionally, acquisition maximization has been used in Bayesian optimization as the optimal decision method. However, we have seen how it can be difficult to parallelize and suffer from model bias. In contrast, acquisition sampling methods can be trivially parallelized, and they are more robust to model mismatch [14].

### 2.1. Acquisition Maximization

Traditionally, query selection in BO involves greedily optimizing an acquisition function α(·) to select the next query $x_{t+1}\in X$, formally:

(5) $$x_{t+1}=\arg\max_{x\in X}\alpha \left(x,p(f\;|\;y_{1:t},x_{1:t})\right)$$

Acquisition functions are designed to trade off exploitation and exploration. Exploitation involves selecting queries that improve upon the current best or that have a high predictive value $\mu (x_{q})$. Exploration involves selecting queries that reduce model uncertainty or that have high uncertainties $\sigma^{2}\left(x_{q}\right)$, respectively.

Common acquisition functions are simple utility functions, such as Upper Confidence Bound [19] and expected improvement (EI) [2]. Other acquisition functions follow a more information theoretic approach about the optimum for query selection, such as Entropy Search [20], Predictive Entropy Search [21], and Max-value Entropy Search [22], and typically involve more compute-intensive calculations.

#### 2.1.1. Expected Improvement

In this paper, without the loss of generality to the choice of acquisition function, we will focus our analysis on EI [2], a popular choice for acquisition function since it provides good BO efficiency in practice while involving relatively fast computations since it relies on the posterior predictive μ and $\sigma^{2}$ to rate a query point $x_{q}\in X$:

(6) $$EI_{t}\left(x\right)=E_{p(y_{t+1}\;|\;y_{1:t},x_{1:t})}\left[\max(0,y_{t+1}-\rho_{t})\right]=h\left(\frac{\mu \left(x\right)-\rho_{t}}{\sigma (x)}\right)\sigma \left(x\right)$$

where $\rho_{t}=\max\left(y_{1},\ldots ,y_{t}\right)$ is the incumbent optimum at current iteration *t* and h(z)=ϕ(z)+zΦ(z), with ϕ and Φ as the standard normal density and cumulative distribution function, respectively.

**Figure 2:**
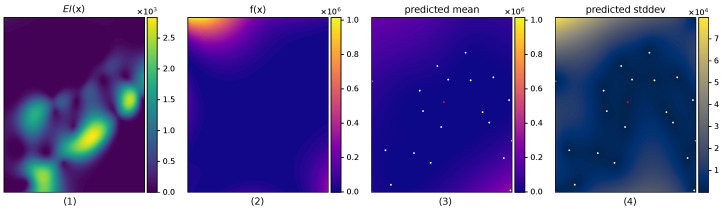
A 2D example showing the multimodality of the acquisition function in the left-most plot. From left to right, it shows (1) the expected improvement EI(x), (2) the function f(x), (3) the predictive mean of the GP μ(x), and (4) the predictive standard deviation of the GP σ(x). Dots represent the observed locations.

Acquisition function optimization is still challenging due to acquisition functions being non-convex. Furthermore, as more data are acquired into the GP, $EI_{t}\left(x\right)$ becomes highly multimodal, which can be seen in Figure 2.

This is a challenge since optimization can be prone to get stuck in local minima. In practice, BO implementations circumvent this by using a multi-restart optimization approach, that is, multiple optimizations are performed from different initial points x∈X with the hope of one reaching the mode of the global optimum.

**Figure 3:**
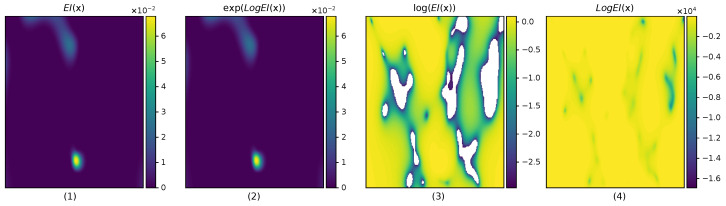
A 2D example problem to show the numerical instabilities of the expected improvement EI(x). It shows different computations of the EI(x) using a double-precision floating point. From left to right, it showcases the inaccuracy of EI(x) compared to LogEI(x): (1) shows the default EI(x), (2) visually shows that LogEI(x) computes the correct quantity of EI(x)≈exp(LogEI(x)), (3) shows the inaccuracies of EI(x) when computing log(EI(x)), with unusable white regions representing minus infinite values resulting from EI(x) being numerically zero, and (4) shows that LogEI(x) implementation is capable of representing EI(x) values much lower than $1\times 10^{-3}$ without numerical issues.

Theoretically, since we use a GP, EI(x) is computed with a Gaussian expectation, $p\left(y|x\right)∼N(\mu \left(x_{q}\right),\sigma^{2}\left(x_{q}\right))$, which has unbounded support, meaning that EI(x) values and gradients are non-zero—although there are some edge cases, such as evaluating at data points locations with zero GP noise for stationary kernels—which is a useful property to help guide the optimization towards higher EI(x) values. However, in practice, when $z=(\mu \left(x\right)-\rho_{t})/\sigma \left(x\right)$ is small, meaning that the current model is very confident that there will be marginally small chances of improvement at x, the EI(x), EI values can become numerically zero. This is a problem when computing the logarithm, as can be seen in Figure 3.

To avoid this, we suggest using the *log expected improvement* function LogEI(x) as presented in [23], which introduces a numerically stable implementation to the logarithm of EI(x). As shown in Figure 3, this is more accurate compared to naively computing log(EI(x)). Since the optimum of log(EI(x)) is the same as that of the EI(x), we can use it interchangeably to find the next query. LogEI(x) can be computed as

(7) $$LogEI\left(x\right)=\log\left(h\left(\frac{\mu \left(x\right)-\rho_{t}}{\sigma (x)}\right)\right)+\log\left(\sigma \left(x\right)\right)$$

where log(h(·)) can be computed in a numerically stable way with

(8) $$\log\left(h\left(z\right)\right)=\left\{\begin{array}{cc} \log(\phi (z)+z\Phi (z)) & z>-1\\ -z^{2}/2-c_{1}+\log1\mathrm{mexp}(\log(\mathrm{erfcx}\left(-z/\sqrt{2}\right)\left|z|\right)+c_{2} & 1/\sqrt{\epsilon }<z\leq -1\\ -z^{2}/2-c_{1}-2\log\left(|z|\right) & z\leq -1/\sqrt{\epsilon } \end{array}\right.$$

where $c_{1}=log\left(2\pi \right)/2$ and $c_{2}=\log\left(\pi /2\right)/2$ and ϵ is the numerical precision. log1mexp and erfcx are numerically stable implementations of log(1−exp(x)) and the *scaled complementary error function* $\exp\left(z^{2}\right)\mathrm{erfc}\left(z\right)$, respectively, and can be found in scientific software libraries, such as the Tensorflow Probability library [24] or SciPy [25].

#### 2.1.2. Thompson Sampling

An alternative approach involves working directly with function values, such as sampling from the posterior distribution of the Gaussian process, drawing i.i.d. samples from the distribution of $\hat{f}∼p\left(f|y_{1:t},x_{1:t}\right)$, and using the maximizer as the next query point:

(9) $$x_{t+1}=\arg\max_{x\in X}\hat{f}\left(x\right)$$

This approach is known as Thompson sampling (TS), a strategy for selecting the next action in a multi-armed bandit problem but, in this case, adapted to the BO setting [4]. Similarly to other acquisition functions, TS is capable of leveraging the exploration and exploitation trade-off. The randomness in the drawn function means that points with high average value (exploitation) and points with high uncertainty (exploration) can yield high values and be the maximizers.

When sampling, $\hat{f}$ is infinite-dimensional; thus, generating exact samples is unfeasible. For computing exact samples, we can use a discretization over the domain X and evaluate samples at those points. However, sampling from a multivariate Gaussian distribution has $O(N^{3})$ computational complexity due to requiring a Cholesky decomposition, which limits the granularity of the discretization. It is beyond the scope of this paper, but note that there are existing approaches to reduce the computation complexity, such as sparse GP approximations [26], leveraging the use of random Fourier features [27] to approximate kernel computations or enabling continuous samples using pathwise conditioning [28].

One of the advantages of TS is that we can parallelize the BO algorithm by drawing multiple functions $\hat{f}$ from the GP, which provides numerous maximizers that we can use to acquire parallel next queries [4].

### 2.2. Acquisition Sampling

**Figure 4:**
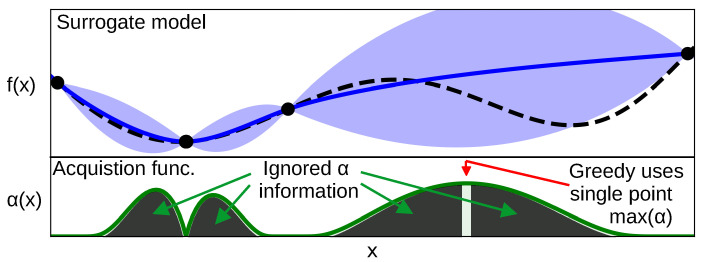
This figure shows a GP surrogate model (blue) plotted on top of an example 1D target function (black dashed). It allows us to showcase that the acquisition function (green) has information across all the input space and, by being greedy and selecting the maximum of the acquisition function (red arrow), we are ignoring a lot of information contained across the domain that can be as valuable as the maximum. Figure inspired by [13].

Selection strategies such as the acquisition functions presented in Section 2.1.1 are spatially greedy as they only select the point that maximizes the acquisition function and ignores all information provided by the acquisition function across the optimization domain X. This can be seen in Figure 4. Moreover, despite acquisition functions being designed to trade off exploration and exploitation to efficiently carry, they rely on the assumption that the surrogate model *is good enough* to predict both the expected function value and its uncertainty accurately. Since, in practice, surrogate model parameters are learned as the BO progresses, model inaccuracies are common, especially at the beginning of the BO, which might impact the theoretical optimality of some acquisition functions.

#### 2.2.1. Boltzmann Sampling

One method that aims to use all the information provided by the acquisition function across the domain and improves model robustness is Boltzmann sampling [13], a sampling approach that uses the Boltzmann policy of the acquisition function:

(10) $$p\left(x_{t+1}\;|\;y_{1:t},x_{1:t}\right)=\frac{e^{\beta_{t}\alpha \left(x_{t+1},p\left(f\;|\;y_{1:t},x_{1:t}\right)\right)}}{\int_{x\in X}e^{\beta_{t}\alpha \left(x,p(f\;|\;y_{1:t},x_{1:t})\right)}dx}$$

This defines a probability distribution for the next query $x_{t+1}∼p\left(x_{t+1}\;|\;y_{1:t},x_{1:t}\right)$. Based on temperature $\beta_{t}$, it allows the model to explore even if the model is completely biased and improves robustness to model inaccuracies [14]. As we will see in Section 3, some methods can work without normalizing the probabilities, which simplifies sampling the next query.

#### 2.2.2. Direct Acquisition Sampling

Boltzmann sampling requires tuning a parameter for the temperature $\beta_{t}$. In practice, this means that this temperature must work throughout the entire Bayesian optimization. This can be challenging due to the variability of the resulting acquisition function as the Bayesian optimization progresses. If we use the EI as an example, at the beginning of BO, due to the sparsity of the data, we can expect higher improvements and thus higher EI values. As BO observes and gets closer to the global optimum, these improvements become smaller. This shows the variability of the EI. Inspired by Boltzmann, we propose carrying the sampling directly on the acquisition function α(·) as an alternative, to avoid tuning $\beta_{t}$, so that the sampling probability becomes

(11) $$p\left(x_{t+1}\;|\;y_{1:t},x_{1:t}\right)=\frac{\alpha \left(x_{t+1},p\left(f\;|\;y_{1:t},x_{1:t}\right)\right)-b}{\int_{x\in X}\alpha \left(x,p(f\;|\;y_{1:t},x_{1:t})\right)dx}$$

with *b* being a bias to ensure strictly positive *p* values to enable sampling when using non-negative acquisition functions. For the EI acquisition function that we will be using, we only need to set b=0.

This formulation has good practical performance, as we will show in our experiments, without having to tune the $\beta_{t}$ parameter. Additionally, a nice property of this formulation is that since we no longer have an exponential, we can introduce the LogEI(x) formulation, which improves the numerical stability of EI(x). This is possible due to sampling methods, such as MCMC, often working with ratios of probabilities, for which, to avoid numerical precision errors, computations are typically carried out using the logarithm of the probability [29]. This improves upon the original Boltzmann sampling [13] with the added benefits of the LogEI(x) computations. Our target log-density probability that we will sample from becomes

(12) $$\log p\left(x_{t+1}\;|\;y_{1:t},x_{1:t}\right)\propto LogEI\left(x_{t+1},p\left(f\;|\;y_{1:t},x_{1:t}\right)\right)$$

As we will see next, some sampling methods do not require the normalization of these log probability values.

## 3. MCMC for Acquisition Sampling

Sampling from an unknown probability typically is performed with a Markov chain Monte Carlo (MCMC) method [15]. The idea behind it is to generate a Markov chain $\left(x_{0},x_{1},\ldots ,x_{n}\right)$ such that the stationary distribution of the chain is the target distribution p(x), that is, as n→∞, it guarantees that $x_{n}∼p\left(x\right)$. In our case, the target distribution is the next query probability $p\left(x\right)=p\left(x_{t+1}\;|\;y_{1:t},x_{1:t}\right)$, as defined in Equations (10) and (11).

MCMC implementations often operate with log densities directly to prevent floating point precision errors. This means that, for acquisition sampling, we can seamlessly introduce LogEI(x), which helps in preventing numerically zero EI(x) values and gradients.

Despite the diverse number of MCMC methods in the literature, sampling an acquisition function for BO presents a unique setting that is not typically found in MCMC applications. First, most acquisition functions are highly multimodal, with low probability areas between modes, as showcased in Figure 2. Second, sampling is performed in a constrained domain because BO operates within fixed bounds, typically a bounding box. In this work, we study different MCMC methods in the acquisition sampling setting and their impact on the resulting performance of the Bayesian optimization method.

### 3.1. Metropolis–Hastings

Metropolis–Hastings (MH) [30] is a classical MCMC method that performs a random walk combined with an acceptance–rejection sampling scheme. Given a current state $x_{n}$, we want to generate $x_{n+1}$ such that it satisfies the MCMC property. For that, it first generates a candidate $x^{\prime }$ from a *proposal distribution*, denoted as $q(x^{\prime }|x_{n})$. Then a ratio of acceptance,

(13) $$r_{a}=\frac{p\left(x^{\prime }\right)q\left(x_{n}|x^{\prime }\right)}{p\left(x_{n}\right)q\left(x^{\prime }|x_{n}\right)}$$

is used to accept or reject the candidate $x^{\prime }$. For u∈[0,1], generated using a uniform, it accepts the candidate $x_{n+1}=x^{\prime }$ if $u\leq r_{a}$, otherwise, it rejects it by setting $x_{n+1}=x_{n}$. Note that, if the proposal *q* is symmetric, this simplifies to $r_{a}=p\left(x^{\prime }\right)/p\left(x_{n}\right)$.

For applying MH in a constrained domain, the general approach is to automatically reject the new candidate $x^{\prime }$ if it lies outside the domain X, that is, we assume that the target probability outside the domain is zero. For dealing with the high multimodality of α(·), we need to carefully select a proposal distribution *q*, since it needs to propose big transitions to jump between modes and smaller transitions so that the acceptance rate is high. If needed, *q* can be defined as mixture distribution, that enables more complex and adapted distributions.

### 3.2. Metropolis-Adjusted Langevin Algorithm

The Metropolis-adjusted Langevin algorithm (MALA) [31] takes steps using a discretized Langevin diffusion, defined by the stochastic differential equation

(14) $$dx_{t}=\frac{1}{2}\nabla_{x}\log\left(p\left(x_{t}\right)\right)dt+dB_{t}$$

where *B* denotes a *d*-dimensional Brownian motion, where in our case *d* corresponds to the problem dimensionality of the inputs. For discrete-time MCMC, a first-order Euler discretization is used:

(15) $$x_{n+1}=x_{n}+\frac{\epsilon^{2}}{2}\nabla_{x}\log\left(p\left(x_{n}\right)\right)+\epsilon u^{n}$$

where u∼N(0,I) and ϵ is the integration step size. To correct first-order integration errors from discretization, the MH acceptance $r_{a}$ is used. In this case, the proposal distribution is $q\left(x_{n+1}|x_{n}\right)∼N\left(\mu ,\sigma^{2}\right)$, where $\mu =x_{n}+\frac{\epsilon^{2}}{2}\nabla_{x}\log\left(p\left(x_{n}\right)\right)$ and $\sigma^{2}=\epsilon^{2}$.

### 3.3. Hamiltonian Monte Carlo

Hamiltonian Monte Carlo (HMC) [32] is a particular case of MH that uses the gradients of the density function to improve the acceptance ratio. It uses an approximate Hamiltonian dynamics simulation with leapfrog numerical integration alongside a Metropolis acceptance step. The idea is to simulate the trajectory of a particle in a potential field defined by the target distribution. It introduces an auxiliary momentum variable ρ to the state and draws from a joint density

(16) p(ρ,x)=p(ρ|x)p(x)

that defines the following Hamiltonian

(17) H(ρ,x)=−logp(ρ|x)−logp(x)

which represents the total energy of the system *H*, with the resulting two log terms often described as the kinetic and potential energy of the system, respectively.

New state candidate $\left(\rho^{\prime },x^{\prime }\right)$ is generated by first drawing momentum $\rho^{\prime }∼N\left(0,M\right)$, where *M* is the mass matrix, and then $\left(\rho^{\prime },x_{n}\right)$ is run under Hamilton’s equations. To solve these motion equations, a symplectic integrator is needed, a numerical integrator for Hamiltonian systems that preserves the Hamiltonian energy. Furthermore, being a particular case of MH, it needs a time-reversible integrator. We will use a leapfrog integrator [15], which is a second-order time-reversible and symplectic integrator. Given a time discretization step size ϵ, it simulates the exact trajectory as

(18) $$\begin{array}{cc} \rho_{t+\epsilon /2} & =\rho_{t}-\frac{\epsilon }{2}\nabla \log p\left(x_{t}\right) \end{array}$$

(19) $$\begin{array}{cc} x_{t+\epsilon } & =x_{t}+\epsilon \rho_{t+\epsilon /2} \end{array}$$

(20) $$\begin{array}{cc} \rho_{t+\epsilon } & =\rho_{t+\epsilon /2}-\frac{\epsilon }{2}\nabla \log p\left(x_{t+\epsilon }\right) \end{array}$$

which updates the momentum for half a step $\rho_{t+\epsilon /2}$, then performs a full step update of position $x_{t+\epsilon }$ using the updated momentum and, finally, the momentum final half step $\rho_{t+\epsilon }$ is updated using the new position. At the end of the leapfrog integration steps, it generates a candidate $\left(\rho^{\prime },x^{\prime }\right)$ that is subjected to an MH acceptance such that $r_{a}=\exp\left(H\left(\rho ,x_{n}\right)-H\left(\rho^{\prime },x^{\prime }\right)\right)$.

### 3.4. No U-Turn Sampler

No U-turn sampler (NUTS) [33] is an HMC method that performs multiple steps informed by first-order gradient information but, as opposed to HMC, it uses a “no U-turn” criterion when sampling trajectories, that is, if the trajectory loops back and begins retracing itself, it stops expanding the trajectory. This improves efficiency by avoiding unnecessary computations and enables an adaptive trajectory length.

### 3.5. Cyclical SGLD

Stochastic gradient Langevin dynamics (SGLD) is a sampling method that combines stochastic gradient descent (SGD) and Langevin dynamics to achieve faster chain convergence. It uses stochastic gradient descent to approximate the gradient calculation; where there is no closed form, it is estimated from a dataset. In this case, we could use SGD to compute the gradient of the GP with a minibatch of the dataset, which could result in a reduced computation load. However, for comparison, we have decided to use the closed-form derivative of the full GP as in the other methods. Thus, the method becomes similar to the MALA. Another advantage of SGLD is that it uses a schedule on the step size such that $\sum_{n=1}^{\infty }\epsilon_{n}=\infty$ and $\sum_{n=1}^{\infty }\epsilon_{n}^{2}<\infty$, which guarantees convergence in probability without the need for the MH acceptance $r_{a}$, as in the MALA.

**Figure 5:**
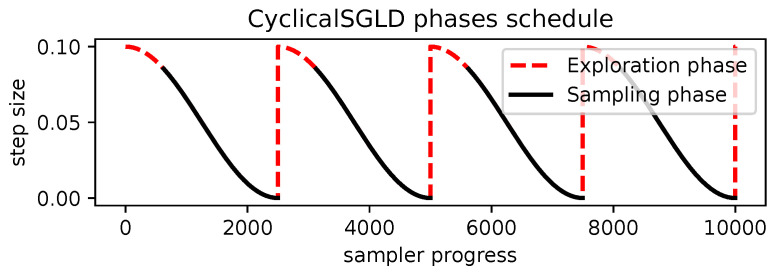
CyclicalSGLD schedules the parameter step size in cycles as the sampler progresses. It shows its two distinct phases: exploration using SGD and sampling using SGLD. Figure inspired by Blackjax’s *The Sampling Book Project* [35].

Furthermore, for multimodal distributions, SGLD can be extended to allow the chain to move between different modes while maintaining the same convergence capabilities. This is called cyclical stochastic gradient Langevin descent (CyclicalSGLD) [34], which extends SGLD by introducing warm restart cycles. For each cycle, it restarts the step size to an initial value and then lowers the step size as discussed before. This process alternates between exploration and sampling stages within each cycle, as shown in Figure 5. Exploration involves stochastic gradient descent (SGD), with a larger step size allowing jumping between modes of the distribution. The sampling stage involves SGLD with a lower step size to properly characterize the local density of the target distribution.

## 4. Results

### 4.1. Methodology

We will evaluate the methods presented in previous sections: *expected improvement maximization* (MaxEI), *Thompson sampling* (TS), and *direct acquisition sampling* (AS). The AS is broken down into different MCMC sampling methods that we evaluate. The analysis will be performed for parallel queries similar to [13], that is, we perform multiple queries per GP update for TS and AS methods. We have not included *Boltzmann sampling* (BS) in the overall comparison as the performance was consistently worse than AS for all the experiments, as shown in Section 4.2.3. Both AS and BS use the expected improvement as the underlying acquisition function.

All methods are implemented using Python 3.10 [36], Jax 0.4.27 [29], and Blackjax 1.2.3 [35] for MCMC implementations and tinygp 0.3.0 [37] for Gaussian processes. Methods relying on gradients use automatic differentiation and parallel queries are computed using automatic vectorization.

All methods use a Gaussian process (GP) with a Matérn kernel with ν=5/2 and additive Gaussian noise $\sigma_{n}=1\times 10^{-3}$.

Initial hyperparameters of the GP are the amplitude scaler $\sigma_{f}=0.1$, lengthscale l=1.0, and mean μ=0, which are then updated by optimizing the log marginal likelihood of the GP. Starting from 10 initial points, each method performs 150 observations. All methods use common random numbers, and experiments are repeated 10 times. Internally, the input domain for each benchmark is normalized to ${\left[0,1\right]}^{d}\in R$, which alleviates the need to choose different parameters for initial PG and method parameters across the different functions.

MaxEI and AS methods use the LogEI(x) numerically stable computation [23]. TS and AS will propose a batch of five queries since its main application is in parallelization and following the experimental setup of [13].

For AS methods, we will evaluate each of the MCMC algorithms introduced within Section 2.2.1. All methods use chains of 4000 length. Note that, in AS methods, the chain length is not equivalent to the number of acquisition evaluations since methods can use multiple chains or perform multiple evaluations for the leapfrog integrator.

The specific parameters for each method are detailed next:

1. Hamiltonian Monte Carlo (HMC) uses a step size of $1\times 10^{-2}$, with five integration steps and identity mass matrix $M=I^{D}$.
2. The Metropolis-adjusted Langevin algorithm (MALA) uses a step size of $1\times 10^{-2}$.
3. No U-turn sampler (NUTS) uses a step size of $1\times 10^{-2}$ and identity mass matrix $M=I^{D}$.
4. CyclicalSGLD uses 30 cycles, alternating exploration and sampling, with a 25–75% ratio, respectively, an initial step size of $1\times 10^{-3}$, and an SGD learning rate of $1\times 10^{-4}$.
5. Mixture distribution Metropolis–Hastings (MMH) uses a proposal that combines different sizes of normal distributions:
  (21) $$q\left(x_{n}\right)=\frac{1}{4}\left(N\left(x_{n},0.01\right)+N\left(x_{n},0.1\right)+N\left(x_{n},0.3\right)+U^{d}\left(0,1\right)\right)$$
  which can be sampled following a stratified sampling strategy:
  (22) $$x_{n}∼\left\{\begin{array}{cc} N(x_{n},0.01) & u<0.25\\ N(x_{n},0.1) & 0.25<u\leq 0.5\\ N(x_{n},0.3) & 0.5<u\leq 0.75\\ U^{d}\left(0,1\right) & 0.75<u\leq 1.0 \end{array}\right.$$
  where u∼U(0,1) is the selecting variable. This proposal achieves a good balance between modeling the locality of the density and jumping between different modes of the acquisition function, considering that the input space is normalized.

To acquire *N* queries from MCMC, there are two practical approaches found in the literature. One involves maintaining a single long chain and uniformly selecting *N* samples from the chain [38]. The other approach involves running multiple independent chains [39], where the chains can be used for convergence analysis, and allowing the chains to be run in parallel. For our implementation, we will rely on the second approach by running *N* independent chains and taking a single sample after a burn-in period of 4000 samples in the chain. The reason is that we can use automatic vectorization to parallelize both the chains and the computations of the target probabilities.

**Figure 6:**
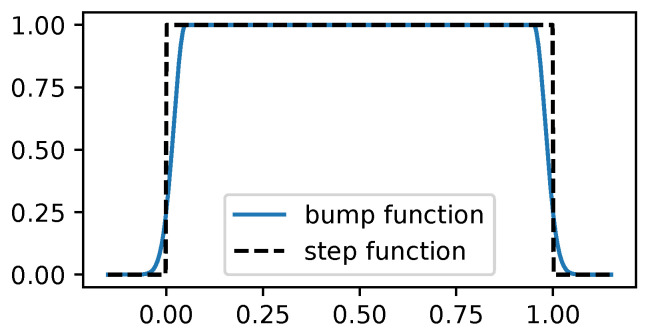
Comparison between a step function and its smoothed-out counterpart using a bump function in the domain (0,1). The advantage of the bump function is that it is derivable and the resulting gradients point back towards the optimization domain. Note that for this visualization, the smooth effect is wider for illustrative purposes. In practice, the width of the smoothness is tighter.

To help prevent any MCMC method from going out of bounds, we drew inspiration from [40], which proposes an unbounded BO with bounds dynamically increasing as needed. To reflect the current boundary in the acquisition function, they put a smooth weighting function over the acquisition function that tends to zero as it approaches the current boundary. In a similar fashion, we propose to smooth the bounds by using

(23) $$w\left(x\right)=a\exp\frac{-x^{2}}{2c^{2}}$$

with parameters a=1 and $c=1\times 10^{-2}$; w(x) can be used to smooth a step function using the bounds of the optimization search, which maintains derivability and helps the gradient to point back towards the search domain, which is useful to prevent MCMC methods from going out of bounds. Figure 6 shows a step function and its equivalent bump function using w(x).

### 4.2. Experiments

Experiments are carried out using benchmark functions typically used in Bayesian optimization [41]. This allows us to know the global optima to better assess the performance of different selection methods and, in particular, the different MCMC methods. The figures show the progress of the incumbent, that is, the current best point found at each optimization step *t*. Since experiments are run multiple times, in the plots, we show the average and a shaded region corresponding to two times the standard deviation of the incumbents across the 10 repetitions. For ease of visualization, we also show the logarithm of the incumbent with respect to the global minima, helping discern the best-performing methods visually. In addition, the legend of each plot is sorted in terms of the performance ranking.

In the following subsections, we study experiments performed on benchmark functions, such as a smooth valley-shaped 2D function and multiple N-dimensional functions, and a real robot problem of an autonomous rover finding the optimal trajectory in uneven terrain.

#### 4.2.1. Benchmark Functions

**Figure 7:**
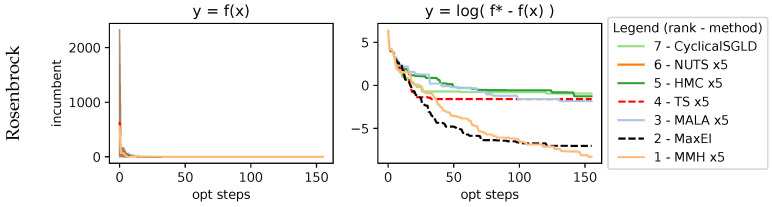
Results on 2D Rosenbrock function. It is a simple function to explore and quickly acquire good-enough values, but the finer exploitation towards optimum is harder, as shown in the log plot. MMH, despite its simplicity, is capable of being as performant as a sequential MaxEI.

The Rosenbrock function [42] is a 2D problem where the optimum lies within a very plain valley. Finding this valley with exploration is easy; however, identifying the global solution is harder. It has the following equation:

(24) $$f_{\mathrm{Rosenbrock}}\left(x\right)=100{\left(x_{2}-x_{1}^{2}\right)}^{2}+{\left(x_{1}-1\right)}^{2}$$

with the function being evaluated within the domain $\left\{x_{1},x_{2}\right\}\in \left\{\left[-0.5,3\right],\left[-1.5,2\right]\right\}$. As shown in Figure 7, it is easy to explore with BO and arrive at the valley, as shown by all methods finding good values very quickly. However, if we look at the logarithmic, it shows the improvements during the exploitation, with MMH finding more refined solutions.

We also evaluate using N-dimensional benchmark functions, that is, functions that are also parametrized by the number of dimensions *d*. This allows us to study the behavior of different methods as dimensionality increases. In particular, we will focus on functions showcasing many local minima, such as Ackley and Alpine.

**Figure 8:**
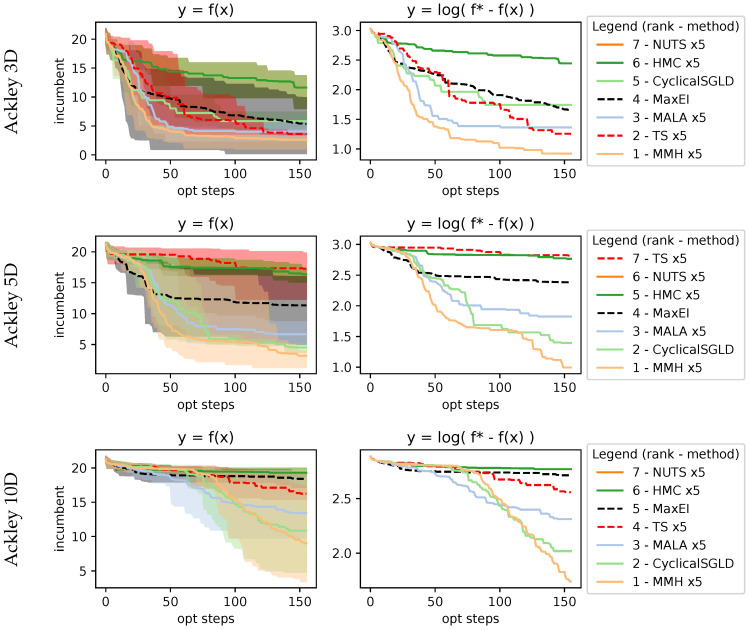
Results on 3D, 5D, and 10D Ackley function. As dimensions increase, optimizing the function becomes more of a challenge; as we can see, sampling methods perform better in higher dimensions.

The Ackley function [42] is a function that contains many local minima. It has the following equation:

(25) $$f_{\mathrm{Ackley}}\left(x\right)=-a\exp\left(-b\sqrt{\frac{1}{d}\sum_{i=1}^{d}x_{i}^{2}}\right)-\exp\left(\frac{1}{d}\sum_{i=1}^{d}\cos cx_{i}\right)+a+\exp\left(1\right)$$

with recommended variables a=20, b=0.2, and c=2π and evaluated within the domain $x_{i}\;\in \left[-32.768,32.768\right]$, and we evaluated it for the 3D, 5D, and 10D cases. Results in Figure 8 show that as dimensions increase, the performance of MaxEI and TS deteriorates compared to AS. Within the AS methods, MMH, CyclicalSGLD, and MALA methods show better performance.

**Figure 9:**
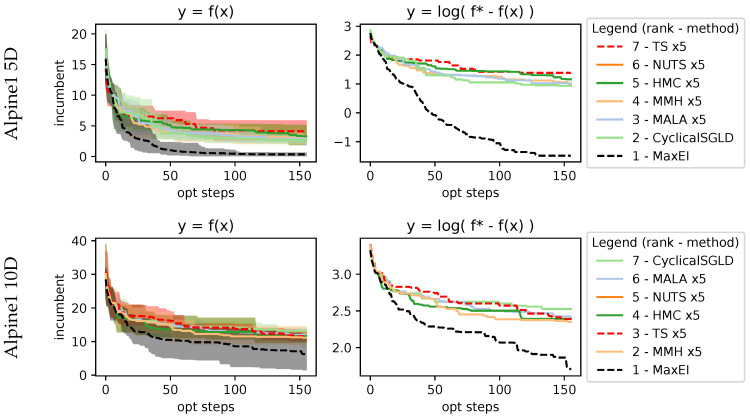
Results on 5D and 10D Alpine1 function. Most sampling methods perform similarly, with minor differences. Sequential maximization in MaxEI performs much better than the rest of the methods due to operating on a 5-query parallelization, which, in theory should always be suboptimal compared to a sequential approach.

**Figure 10:**
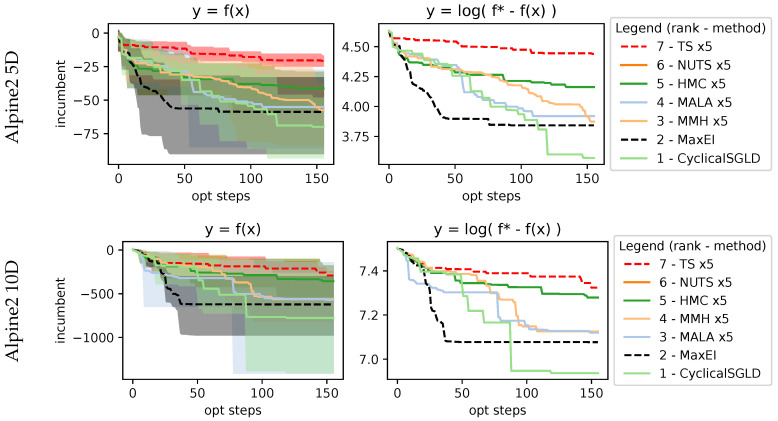
Results on 5D and 10D Alpine2 function. This function shows acquisition sampling capable of exploring much better than function sampling done by TS.

We also evaluate the Alpine family of functions [41], which are two functions that also have many local minima. They have the following equations:

(26) $$\begin{array}{cc} f_{\mathrm{Alpine}1}\left(x\right) & =\sum_{i=1}^{d}\left|x_{i}\sin\left(x_{i}\right)+0.1x_{i}\right| \end{array}$$

(27) $$\begin{array}{cc} f_{\mathrm{Alpine}2}\left(x\right) & =\prod_{i=1}^{d}\sqrt{x_{i}}\sin\left(x_{i}\right) \end{array}$$

with the search space being $x_{i}\in \left[-10,10\right]$ and $x_{i}\in \left[1,10\right]$ respectively. Alpine1 evaluation at 5D and 10D in Figure 9 shows how MaxEI performs better. For AS methods, only CyclicalSGLD matches the performance of MaxEI. MMH is the second, followed by the MALA. However, it seems that methods are stuck in suboptimal regions. This can be seen in Alpine2, evaluated at 5D and 10D, shown in Figure 10, which has a smaller search space and is able to reach better incumbent values. In this case, the best-performing method is CyclicalSGLD, followed by the MALA and MMH.

#### 4.2.2. Rover Trajectory Problem

**Figure 11:**
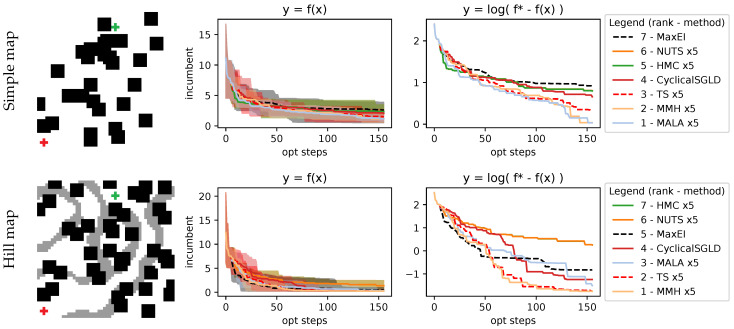
Results on the rover trajectory problem, showing a simple map and a map with hills. The starting position of the trajectory is from the red cross at the lower left, and the goal is the green cross at the top side. The cost of traversing a terrain is determined by how dark the space is in the picture, with zero cost in white. Results show that AS and TS are capable of finding the zero-cost trajectory, with MMH and the MALA clearly showing better performance than all the AS methods.

In this section, we cover the problem of finding the lowest-cost trajectory of a rover in a surface map between an initial point and an endpoint, where the cost represents possible terrain irregularities or obstacles that we wish to avoid. This setting is based on *optimizing rover trajectories* from Wang et al. [43], and we follow the changes introduced in [14]. We define a parametrized trajectory with a B-spline that starts fixed at the starting position, ends fixed at the goal position, and, as parameters, uses two control points that will define the curvature of the B-spline. The overall dimensionality of the problem we are solving is 4D since we have to optimize the two control points that are 2D. Trajectory cost is integrated along the trajectory at a fixed interval using 1000 steps. We use two different maps, as shown in the picture inside Figure 11, with both maps sharing the same start and goal but differing on the costs (e.g., different obstacles). Results are also shown in Figure 11, where it shows that MaxEI has some trouble finding the optimal zero-cost trajectory. Meanwhile, AS and TS are capable of finding such a trajectory. In particular, MMH and the MALA are the AS methods that are capable of solving the problem.

#### 4.2.3. Ablation on Boltzmann Sampling

**Figure 12:**
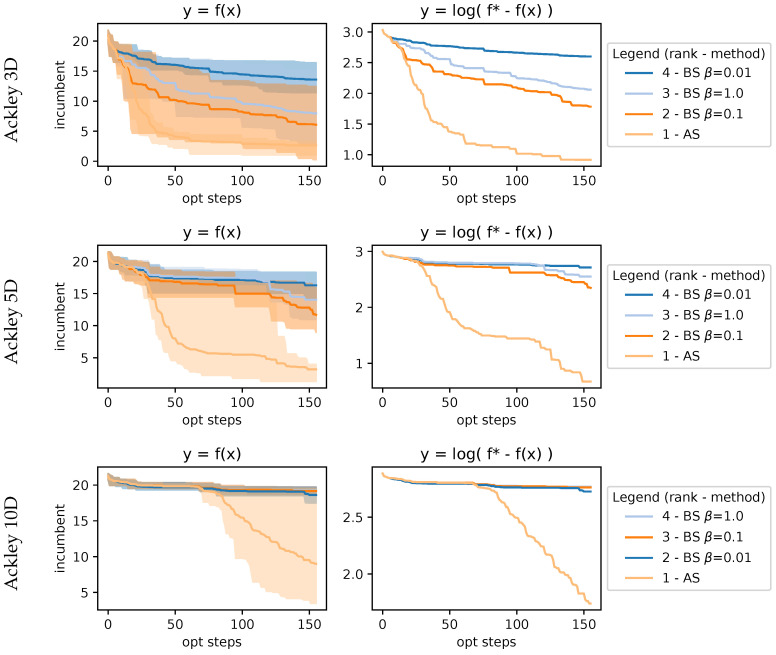
Results on 3D, 5D, and 10D Ackley function using MMH comparing AS against BS with different temperatures. These plots showcase the difficulty of tuning the β temperature parameter for BS. It shows how AS performs better without a parameter.

Here we compare Boltzmann sampling (BS) with different temperatures to direct acquisition sampling (AS). We compared them using the same MMH sampling method, which we chose since it was the best-performing MCMC method overall. The results in Figure 12 showcase how difficult is to choose a good fixed temperature for BS. We can clearly see that there is a point in the BO wherein the BS methods underperform, which aligns with our hypothesis that a single fixed temperature is not capable of working properly throughout the optimization, whereas our proposed approach AS circumvents this limitation. We only show results on the Ackley function, but the results were consistent in the other benchmarks.

## 5. Discussion

The proposed experiments focused on studying the different MCMC methods when sampling from the acquisition function. Overall, out of the methods that we evaluate, CyclicalSGLD, MMH, and the MALA are the only methods that consistently rank on top.

The most underperforming methods, NUTS and HMC, suffer from one of the challenges of acquisition sampling, that is, the variability in the function that we sample as the BO progresses and its relationship to the MCMC parameters. In particular, if the mass matrix *M* is a poor approximation of the target distribution covariance, it will be required to compensate for other parameters to perform a more fine-grained integration (i.e., smaller step size ϵ and a higher number of integration steps) to maintain numerical precision. Even when actively tuning these parameters, it is still challenging [44]. In our setup, failing to choose good parameters across all Bayesian optimization steps leads to poor performance. Out of the best-performing methods, CyclicalSGLD and the MALA share that they use the gradient information, helping to improve MCMC efficiency, and the parameters are not as critical for chain convergence or hard to choose. Finally, the most interesting result is the MMH. Metropolis–Hastings is a classic algorithm that is also quite simple compared to other methods since it only has a single parameter: the proposal distribution. In our experiments, we opted for a mixture of distributions as a proposal, with a uniform distribution allowing global jumps to change between multiple modes of the acquisition function and multiple Gaussian processes that enable traversing the locality of the probability. Experiments show that, even on higher dimensions, this mixture approach is performant.

Regarding the bounded problems, our bump function helps to keep the chain within the search space of BO. However, with high gradients, the chain might jump out of bounds directly and reject many states of the MCMC chain. As a takeaway, we propose MMH as the better alternative, since we can reject jumps out-of-bounds, avoiding the problems associated with the previous methods.

Future research of this work will involve the bounded setting of MCMC methods, which, in the context of MCMC, is quite limited in the literature, especially if we focus on highly multimodal densities. In particular, research will include modifying good-performing methods, such as the MALA and CyclicalSGLD, to include bounds or complement with methods that actively deal with bounded domains, such as hit-and-run methods [45]. Additionally, automatic tuning of the MCMC parameters using the initial *N* samples as burn-in could be used to improve some of the methods’ overall performance or adapt the parameters using an inner loop of Bayesian optimization [44].

## Data Availability

The data presented in this study are openly available on GitHub at https://github.com/jgbarcos/better_bo_sampling, accessed on 7 January 2025.

## Abbreviations

The following abbreviations are used in this manuscript:

BO	Bayesian optimization
GP	Gaussian process
EI	Expected improvement
LogEI	Log expected improvement
MaxEI	Maximization expected improvement
TS	Thompson sampling
BS	Boltzmann sampling
AS	Direct acquisition sampling
MCMC	Markov chain Monte Carlo
MH	Metropolis–Hastings
MMH	Mixture distribution Metropolis–Hastings
MALA	Metropolis-adjusted Langevin algorithm
HMC	Hamiltonian Monte Carlo
NUTS	No U-turn sampler
SGLD	Stochastic gradient Langevin dynamics
SGD	Stochastic gradient descent
